# Long Non-Coding RNA-PAICC Promotes the Tumorigenesis of Human Intrahepatic Cholangiocarcinoma by Increasing YAP1 Transcription

**DOI:** 10.3389/fonc.2020.595533

**Published:** 2021-01-08

**Authors:** Long Xia, Xiaolong Chen, Jiarui Yang, Shuguang Zhu, Lei Zhang, Qi Yin, Yueyu Hong, Haoqi Chen, Guihua Chen, Hua Li

**Affiliations:** ^1^ Department of Hepatic Surgery and Liver Transplantation Center, The Third Affiliated Hospital, Sun Yat-Sen University, Guangzhou, China; ^2^ Department of Guangdong Key Laboratory of Liver Disease Research, The Third Affiliated Hospital, Sun Yat-Sen University, Guangzhou, China; ^3^ Department of Hepatobiliary Surgery, The Eighth Affiliated Hospital, Sun Yat-Sen University, Shenzhen, China; ^4^ Department of Biliary-Pancreatic Surgery, The Third Affiliated Hospital, Sun Yat-Sen University, Guangzhou, China; ^5^ Department of Project, CookGen Biosciences Center, Guangzhou, China; ^6^ Department of Bioinformation, Forevergen Biosciences Co., Ltd, Guangzhou, China

**Keywords:** lncRNAs, intrahepatic cholangiocarcinoma, ceRNA, MiR-27a-3p, MiR-141-3p, Hippo pathway

## Abstract

Intrahepatic cholangiocarcinoma (ICC) is a heterogeneous hepatobiliary tumor with poor prognosis, and it lacks reliable prognostic biomarkers and effective therapeutic targets. Long non-coding RNAs (lncRNAs) have been documented to be involved in the progression of various cancers. However, the role of lncRNAs in ICC remains largely unknown. In the present work, we used bioinformatics analysis to identify the differentially expressed lncRNAs in human ICC tissues, among which lncRNA-PAICC was found to be an independent prognostic marker in ICC. Moreover, lncRNA-PAICC promoted the proliferation and invasion of ICC cells. Mechanistically, lncRNA-PAICC acted as a competitive endogenous RNA (ceRNA) that directly sponged the tumor suppressive microRNAs miR-141-3p and miR-27a-3p. The competitive binding property was essential for lncRNA-PAICC to promote tumor growth and metastasis through activating the Hippo pathway. In summary, our results highlighted the important role of the lncRNA-PAICC-miR-141-3p/27a-3p-Yap1 axis in ICC, which offers a novel perspective on the molecular pathogenesis and may serve as a potential target for antimetastatic molecular therapies of ICC.

## Introduction

Intrahepatic cholangiocarcinoma (ICC) is the common liver malignant tumor after hepatocellular carcinoma (HCC). In the past 40 years, the global morbidity and mortality of ICC have increased significantly, highlighting the increasing clinical challenge that was once regarded as a rare disease (1). In recent years, despite advances in ICC standards of care and management options, the prognosis of this devastating cancer is still poor. The poor prognosis and high recurrence rate of ICC is largely due to its high invasiveness and high rate of early stage metastasis. At present, surgical resection is the sole curative treatment option to achieve long-term survival. However, only approximately 30–40% of ICC patients have the opportunity to receive surgery, and the 5-year survival rate is only 20–40%. For patients with unresectable and recurring ICC, standard chemotherapy regimens (gemcitabine and cisplatin) are only palliative treatments, resulting in limited survival benefits (2, 3). In contrast to hepatocellular carcinoma, no specific targeted molecular therapy for ICC has been approved so far, and limited clinical trials of targeted therapy for ICC have also ended in failure (4, 5), thereby emphasizing the urgency of identifying new therapeutic targets for successful intervention.

The Hippo pathway is an important regulator of cell growth, apoptosis, and tumorigenesis, and this pathway is highly conserved in mammals (6). The components of the Hippo pathway include MST1/2, Sav1, LATS1/2, MOB1, and YAP1. YAP1 is the main downstream effector molecule of the pathway, and it can be phosphorylated directly by LATS1/2. Phosphorylated YAP1 binds to proteins in the cytoplasm and is degraded by ubiquitination, thus inhibiting the growth-promoting and anti-apoptosis functions of YAP1. The upstream signal elements in the Hippo pathway are suppressors, while YAP1 is negatively regulated by the signal cascade and is considered to be the oncogene in several cancers (7). However, the current research on the role and mechanism of this signaling pathway in ICC remains unclear.

Cancer is a genetic disease that interrupts cell homeostasis and promotes uncontrollable growth by changing the flow of cell information (8). The discovery of the genetic code of encoding proteins has brought a major breakthrough in understanding how gene mutations cause cancer, and it has established scientific principles for targeted therapies (9, 10). However, aberrations within non-coding genomes lead to cancer phenotypes. One of the most striking discoveries of the genome era was the recognition that RNA is widely transcribed from non-protein coding regions because the coding genome accounts for less than 2% of all sequence. At first, lncRNA was considered as the “noise” of genome transcription and had no biological function. Further research has found that lncRNA is a type of non-coding RNA that has tissue expression specificity and can participate in diverse biological and pathological processes (11, 12). Many studies have reported that lncRNAs drive many important cancer phenotypes (13–19). However, little attention has been focused on identifying and elucidating the mechanisms of lncRNAs and their influence on the invasion-metastasis cascade in ICC.

The advent of the era of high-throughput sequencing and data mining has provided tremendous insights into the genome landscape and has greatly facilitated the discovery of new valuable biomarkers in the field of cancer (20). In this study, we elucidated the ceRNA network of ICC through data mining. We identified lncRNA-RP11-375I20.6 (Gene ID: ENSG00000260633), which we named as prognostic-associated ICC lncRNA (lncRNA-PAICC), and more importantly, we found that it is critical in ICC tumorigenesis. As a ceRNA, lncRNA-PAICC competitively binds to miR-141-3p and miR-27a-3p. The competitive action of lncRNA-PAICC is essential to activate the Hippo pathway and thus facilitate the invasion-metastasis cascade. Our research presents new insights for understanding the mechanism of ICC and exploring potential therapeutic targets.

## Materials and Methods

### Bioinformatics Analysis of Data Sets

We searched the GEO database and obtained the following two data sets: GSE107943 and GSE53870. The GEPIA database (http://gepia.cancer-pku.cn/index.html) is a comprehensive online tool that integrates TCGA cancer big data and GTEx normal tissue projects. The database uses bioinformatics technology to solve important problems in cancer biology, revealing cancer subtypes, driving genes, differential expression or carcinogenic factors to explore new cancer targets and biomarkers.

### Identification of ceRNA Regulatory Network Involved in Intrahepatic Cholangiocarcinoma

First, we analyzed the GSE107943 data set to identify significantly differentially expressed lncRNAs and mRNAs, and we then performed coexpression and gene function enrichment analysis to elucidate the main biological processes and signal pathways involved and further verify and screen the differentially expressed genes (DEGs) through the GEPIA database. Second, we analyzed the microRNAs expression profile of GSE53870 to acquire differentially expressed microRNAs and further predicted their target genes through online databases (miRTarBase, TargetScan and DIANA Tools). Finally, based on the regulatory relationship of the ceRNA network, we integrated the key regulatory relationship to establish the eventual ceRNA coexpression network.

The differential expression of lncRNAs and mRNAs in ICC and adjacent tissues was detected by a t-test. The correlation between differentially expressed lncRNAs and mRNAs expression was calculated by Spearman’s correlation test. The final ceRNA network was screened and determined according to the following criteria: 1) length of lncRNAs between 200 and 2500 bp; 2) at least two binding sites between lncRNAs and miRNAs; 3) increase in lncRNA expression is correlated to poor prognosis; and 4) decrease of miRNA expression is associated with unfavorable prognosis. R 3.5.2 (https://www.r-project.org/) was used to perform all analyses.

### Patients and Clinical Samples

From January 2014 to December 2019, 76 pairs of fresh intrahepatic cholangiocarcinoma and corresponding adjacent samples were collected from the Third Affiliated Hospital of Sun Yat-sen University (part of which was from the Clinical Specimen Bank of the Third Affiliated Hospital of Sun Yat-sen University). All patients had no preoperative intervention or radiotherapy. All patients in this study signed an informed consent form and were approved by the Institutional Ethical Board of Third Affiliated Hospital of Sun Yat-sen University. Postoperative follow-up of patients was followed by outpatient clinic and telephone follow-up every 3 months until the patient had disease-related death or the study was terminated.

### Statistical Analysis

Statistical analyses were performed using statistical software R (version 3.5.2; http://www.r-project.org) and GraphPad Prism (version 8.0). P <0.05 was defined as statistically significant. Overall survival (OS) curves were calculated with the Kaplan–Meier method and were analyzed with the log-rank test.

### Other Adopted Methods

Other experiments included cell treatments, RT-PCR, subcellular localization, construction of cell vectors, transfection of cell vectors, luciferase reporter assays, RNA immunoprecipitation, MS2-RIP, western blotting, immunohistochemistry and animal studies. Please refer to Appendix File 3 for specific details of these experiments.

## Results

### Construction and Validation of Yes-Associated Protein 1-Related ceRNA Regulatory Networks for Intrahepatic Cholangiocarcinoma

**Figure 1:**
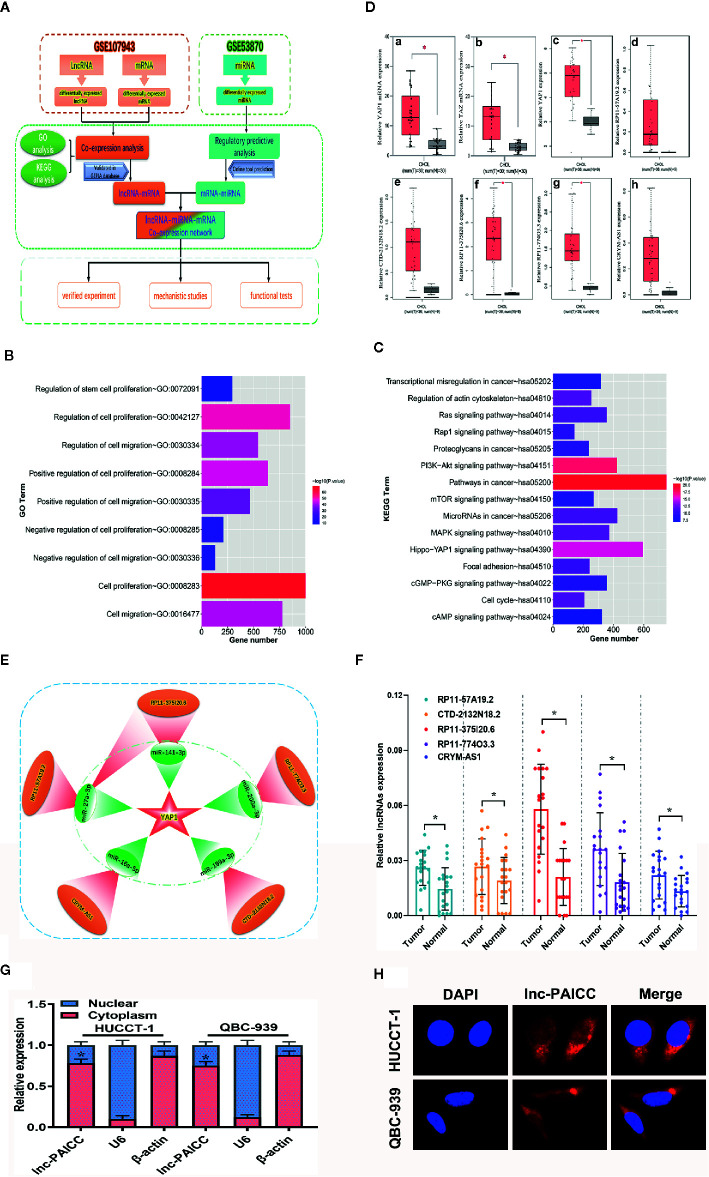
Construction and validation of YAP1-related ceRNA regulatory networks for ICC. **(A)** Bioinformatics Analysis flow diagram. **(B)** GO annotation of differentially expressed genes. **(C)** KEGG analysis of differentially expressed genes. **(D)** (a) The relative expression level of YAP1 in the GSE107943 data set (comparison between cancer and corresponding normal tissue, *P<0.05); (b) The relative expression level of TAZ in the GSE107943 data set (comparison between cancer and corresponding normal tissue, *P<0.05); (c) The relative expression level of YAP1 in the GEPIA database (comparison between cancer and corresponding normal tissue, P < 0.05); c–h show the relative expression of six genes YAP1, lncRNA-RP11-57A19.2, lncRNA-CTD-2132N18.2, lncRNA-RP11-375I20.6, lncRNA- RP11-774O3.3, and lncRNA-CRYM-AS1 in the GEPIA database, respectively. **(E)** The schematic diagram of ceRNA network obtained by bioinformatics analysis. This subnetwork consists of five lncRNA (red) and five corresponding microRNA (green). **(F)** The differential expression of five candidate lncRNAs in 20 paired ICC and normal samples was examined by RT–PCR. Data are mean ± SD (n = 20), *P<0.05. **(G)** RT–PCR analysis of lncRNA-PAICC expression in HUCCT-1 and QBC-939 cells. *β*-actin and U6 were used as endogenous controls. Data are mean ± SD (n = 3). *P < 0.05. **(H)** Subcellular localization of lncRNA-PAICC by RNA-FISH in HUCCT-1 and QBC-939 cells.

We retrieved and obtained the relevant data sets of ICC from the GEO database, and we then analyzed these data sets by bioinformatics (**Figure 1A**). In brief, we analyzed the differentially expressed lncRNAs and mRNAs in GSE107943 and then performed functional enrichment analysis. GO annotation showed that these DEGs were mainly involved in cell proliferation and migration (p < 0.01) and other biological pathways (**Figure 1B**). KEGG analysis revealed that these DEGs were mainly involved in signaling pathways regulating cell proliferation and metastasis (**Figure 1C**), especially the Hippo pathway (p < 0.01). Therefore, we further analyzed the expression of key genes in the Hippo pathway in GSE107943, and we found that the key effector gene, Yes-associated protein (YAP1) and its homologous transcriptional coactivator, TAZ, were significantly elevated in ICC tissues (**Figure 1D**, a–b). At the same time, we verified that the expression of YAP1 was also significantly increased in the GEPIA database compared with that in normal bile duct tissue (**Figure 1D**, c). The above analysis preliminarily confirmed that the Hippo pathway is involved in the progress of ICC. Subsequently, we analyzed the lncRNAs in the above data set that were coexpressed with YAP1, and we also confirmed that the expression level of these lncRNAs was elevated compared to that in normal bile duct tissue in the GEPIA data (P < 0.05) (**Figure 1D**, d–h). By analyzing the GSE53870 data set and combining with online prediction tools (miRTarBase, TargetScan and DIANA Tools), we identified the differentially expressed microRNAs that retarget and regulate YAP1. Finally, by integrating the regulatory relationship, we obtained a YAP1-related ceRNA regulatory network (**Figure 1E**), including five lncRNAs and five corresponding microRNAs.

To further validate the bioinformatics results, the five candidate lncRNAs (RP11-57A19.2, CTD-2132N18.2, RP11- 774O3.3, RP11-375I20.6 and CRYM-AS1) were detected in 20 pairs of ICC and corresponding normal samples by RT-PCR. Compared with those in neighboring tissues, the expression levels of the five lncRNAs were upregulated in ICC tissues, and lncRNA-RP11-375I20.6 had the highest expression level (**Figure 1F**). Therefore, we selected this lncRNA for subsequent studies, and we named it the prognosis-associated intrahepatic cholangiocarcinoma lncRNA (lncRNA-PAICC).

By utilizing LNCipedia (https://lncipedia.org/), an online database integrating multiple databases for human lncRNAs, we obtained the basic annotation information of lncRNA-PAICC with a complete sequence of 1,946 bp (**Supplementary Figures 1A, D**). The coding ability of lncRNA-PAICC was predicted by the following two online coding databases: Coding Potential Calculator (http://cpc.cbi.pku.edu.cn/) and Coding-Potential Assessment Tool (http://sourceforge.net/projects/rna-cpat). These prediction tools adopted different algorithms based on the sequence characteristics of lncRNA-PAICC. Although this lncRNA has a 306 nt open reading frame (ORF) (**Supplementary Figure 1C**), both analyses suggested that it had no coding potential (**Supplementary Figures 1B, C**) with sensitivity and specificity of the coding ability prediction being 0.96 and 0.97, respectively (21). RT–PCR analysis, fluorescence *in situ* hybridization and cytoplasmic/nuclear fractionation indicated that lncRNA-PAICC is mainly located in the cytoplasm (**Figures 1G, H**). Thus, the above data indicated the identification of a novel lncRNA for ICC.

### Novel Long Non-Coding RNA Is Closely Related to Yes-Associated Protein 1 Expression

**Figure 2:**
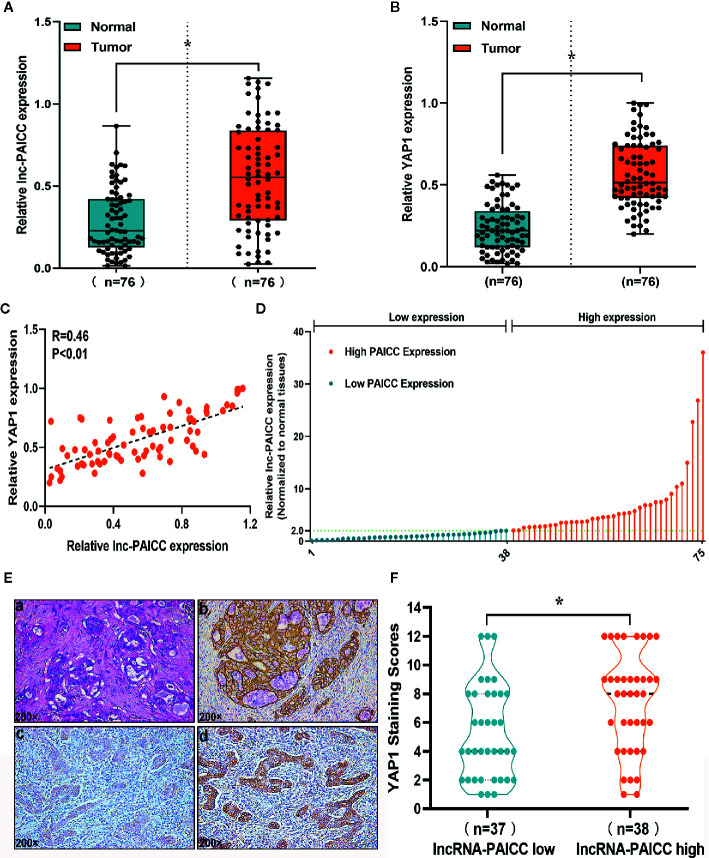
The novel lncRNA is closely related to YAP1 expression. **(A)** The relative expression levels of lncRNA-PAICC in 76 pairs of ICC tumors and corresponding adjacent normal tissues were detected by RT-PCR. Data are mean ± SD (n = 76), *P<0.05. **(B)** The relative expression levels of YAP1 in 76 pairs of ICC tumors and corresponding adjacent normal tissues were detected by RT-PCR. Data are mean ± SD (n = 76), *P<0.05. **(C)** The correlation between lncRNA-PAICC transcript level and YAP1 mRNA transcript level was measured in 76 ICC tissues. The 2^−△△Ct^ values were subjected to Spearman’s correlation analysis. **(D)** The patients were classified into two groups according to relative lncRNA-PAICC expression level. **(E)** Staining of paraffin sections of human ICC tissue. (a) Hematoxylin and eosin-stained images of ICC tissues; (b) CK19 expression in ICC tissue; (c) Weak expression of YAP1 in ICC tissue; (d) High expression of YAP1 in ICC tissue. **(F)** In the low or high expression groups of lncRNA-PAICC, the average staining score of YAP1 was evaluated by the scatter plot. Data are mean ± SD. *P < 0.05.

After confirming that lncRNA-PAICC levels were significantly increased in ICC tissues, we evaluated 76 pairs of ICC tissues and corresponding normal tissues for the expression of lncRNA-PAICC (**Figure 2A**) and YAP1 (**Figure 2B**) by RT-PCR. The expression levels of lncRNA-PAICC and YAP1 in ICC were significantly higher than those in normal tissues. In addition, all 76 ICC tissues were included for Spearman’s correlation analysis, which indicated a significant positive correlation between lncRNA-PAICC and YAP1 (r = 0.46, p < 0.05; **Figure 2C**). These data confirmed that lncRNA-PAICC expression is significantly enhanced in ICC specimens (2.0-fold, **Figure 2A**). The 76 recruited ICC patients were then classified into a high expression group (>2.0) and low expression group (<2.0) (**Figure 1D**). Subsequently, we performed immunohistochemical analysis on ICC paraffin sections in the lncRNA-PAICC high and low expression groups (**Figure 2E**). The results showed that the staining score of YAP1 in the lncRNA-PAICC high expression group was significantly higher than that in the low expression group (**Figure 2F**).

These data indicated that there is a significant correlation between YAP1 and lncRNA-PAICC in ICC tissue.

### Long Non-Coding RNA Expression Level Is Closely Related to the Prognosis of Intrahepatic Cholangiocarcinoma Patients

**Figure 3:**
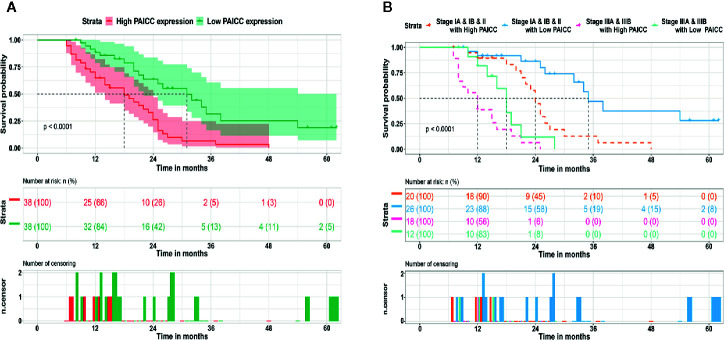
LncRNA-PAICC expression level is closely related to the prognosis of ICC patients. **(A)** The overall survival rates of 76 ICC patients with high and low expression of lncRNA-PAICC were compared. P<0.05, P-value was calculated by log-rank test. **(B)** Kaplan–Meier survival curve analysis of ICC patients with different expression levels of lncRNA-PAICC stratified by ICC TNM stage. P<0.05. P-value was calculated by log-rank test.

After confirmation of elevated lncRNA-PAICC in ICC specimens, we further investigated its clinical implication based on clinical and pathological data. We found that the lncRNA-PAICC expression was related to tumor number (p = 0.022), tumor size (p = 0.034), and vascular invasion (p = 0.001) for ICC patients (**Table 1**). Kaplan–Meier analysis showed that the overall survival (OS) of ICC patients with high expression of lncRNA-PAICC was significantly worse than that of ICC patients with low expression of lncRNA-PAICC (**Figure 3A**). More importantly, when ICC patients were stratified according to TNM stage combined with high and low expression of lncRNA, the OS of patients with low expression of PAICC was significantly better than that of patients with high expression of PAICC (**Figure 3B**). In summary, the above data indicated that the expression level of lncRNA-PAICC is strongly linked to the prognosis of ICC patients.

**Table 1:** Correlation between PAICC expression and clinicopathologic characteristics of ICC patients^a^.

Characteristics	Cases (%)	lncRNA-PAICC expression level		
		High	Low	*p*-value
**Total Cases**	76 (100.00)	38	38	
**Gender**				
Female	32 (42.10)	16	16	1.000
Male	44 (57.90)	22	22	
**Age**				
<=55	28 (36.85)	15	13	0.812
>55	48 (62.15)	23	25	
**Pathological**				
moderately	46 (60.50)	22	24	0.320
poorly	21 (27.60)	13	8	
well	9 (11.90)	3	6	
**CA-199**				
<=35	12 (15.80)	4	8	0.208
>35	64 (84.20)	34	30	
**CA-125**				
<=35	50 (65.80)	25	25	1.000
>35	26 (34.20)	13	13	
**Tumor Number**				
Multiple	11 (14.50)	9	2	0.022*
Single	65 (85.50)	29	36	
**Tumor Size**				
<=5	29 (38.15)	10	19	0.034*
>5	47 (61.85)	28	19	
**Vascular invasion**				
No	41 (53.95)	13	28	0.001*
Yes	35 (46.05)	25	10	
**TNM-T-Stage**				
T1a	18 (23.70)	8	10	0.232
T1b	14 (18.40)	7	7	
T2	34 (44.70)	15	19	
T3	10 (13.20)	8	2	
**Lymphatic metastasis**				
N0	56 (73.70)	26	30	0.297
N1	20 (26.30)	12	8	
**TNM-Stage (AJCC)^b^**				
IA	16 (21.05)	6	10	0.647
IB	14 (18.40)	6	8	
II	16 (21.05)	8	8	
IIIA	10 (13.15)	6	4	
IIIB	20 (26.35)	12	8	

N of cases, number of cases; T stage, tumor stage; TNM, tumor node metastasis.

^a^Chi-square test, *p < 0.05.

^b^American Joint Committee on Cancer (AJCC), patients were staged in accordance with the 8th Edition of the AJCC Cancer’s TNM Classification. The bold values indicate statistically significant differences (P < 0.05).

### Intrahepatic Cholangiocarcinoma-PAICC Promotes Proliferation, Promotes Invasion and Regulates Yes-Associated Protein 1 Expression in Intrahepatic Cholangiocarcinoma Cells *In Vitro*

**Figure 4:**
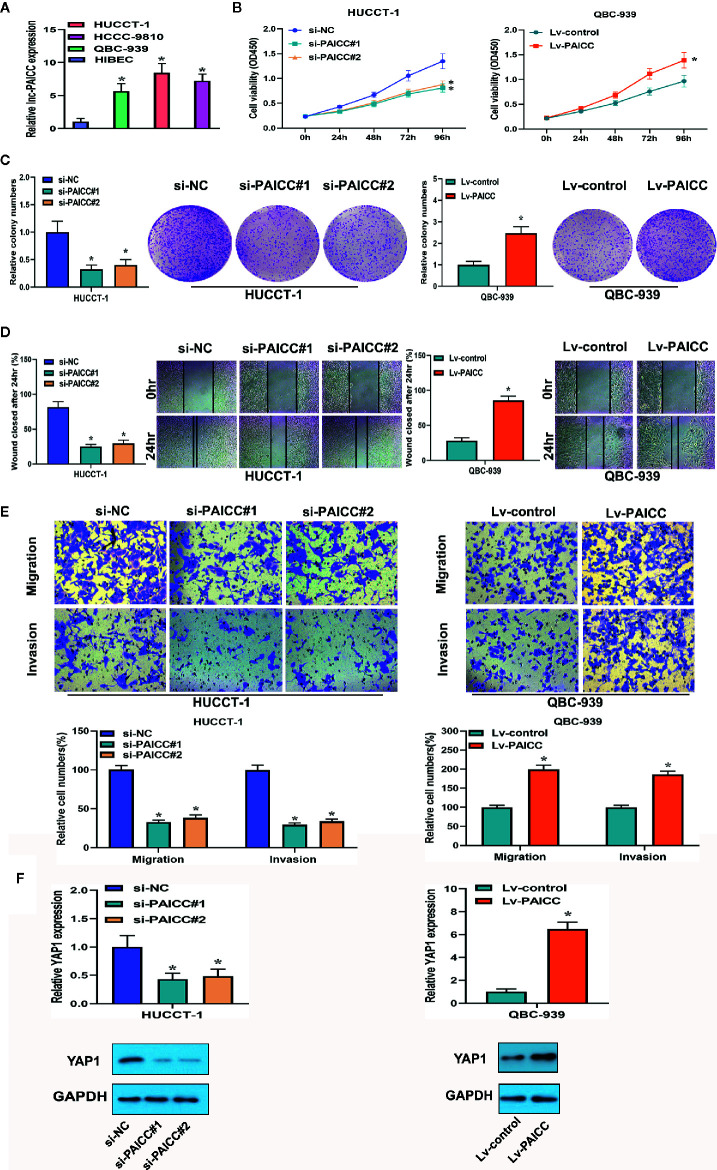
The lncRNA-PAICC promotes proliferation, promotes invasion and regulates YAP1 expression in ICC cells *in vitro.* **(A)** Relative expression of lncRNA-PAICC in ICC cell lines by RT-PCR. **(B)** CCK-8 assays were used to examine cell viability of HuCCT-1 and QBC-939 cells after transfection. **(C)** Colony formation assays were used to detect the clone ability of HuCCT-1 and QBC-939 cells after transfection. **(D)** Wound healing assays were shown in HuCCT-1 and QBC-939 cells after transfection. **(E)** Transwell assays were used to detect cell migration and invasion capacities of HuCCT-1 and QBC-939 cells after transfection. **(F)** RT-PCR and western blotting were used to detect mRNA and protein expression levels of YAP1 in HUCCT-1 and QBC-939 after knockdown and overexpression of lncRNA-PAICC, respectively. **(A**–**F)** The data were Data are mean ± SD. Data between two groups were tested using independent sample t-test. *P < 0.05. All the experiments were repeated three times.

To investigate the biological function of lncRNA-PAICC in ICC cell lines, we detected the expression of lncRNA-PAICC in ICC cell lines and normal human intrahepatic bile duct epithelial cells (HIBECs) by RT-PCR, and we confirmed that lncRNA-PAICC expression in ICC cell lines was significantly higher than that in HIBECs (**Figure 4A**). Subsequently, we stably knocked down lncRNA-PAICC in HUCCT-1 cells due to its relatively high expression and overexpressed lncRNA-PAICC in QBC-939 cells due to its relatively low expression (**Supplementary Figures 2A, B**). Cell-counting kit-8 (CCK-8) assays, colony formation assays, and wound-healing assays all demonstrated that the proliferation of HUCCT-1 cells was significantly inhibited when lncRNA-PAICC was silenced (**Figures 4B–D**, left panel). In contrast, the proliferation of QBC-939 cells was significantly increased after exogenous overexpression of lncRNA-PAICC (**Figures 4B–D**, right panel). Transwell assays showed that lncRNA-PAICC knockdown significantly reduced the migration and invasion ability of HUCCT-1 cells *in vitro* (**Figure 4E**, left panel). In contrast, the migration and invasion ability of QBC-939 cells was dramatically improved after exogenous overexpression of lncRNA-PAICC (**Figure 4E**, right panel). In view of the significant positive correlation between the expression of YAP1 and lncRNA-PAICC, we also showed that silencing of lncRNA-PAICC in HUCCT-1 cells resulted in decreased YAP1 in mRNA and protein levels (**Figure 4F**, left panel), and when lncRNA was overexpressed in QBC-939 cells, the trend was reversed (**Figure 4F**, right panel).

### Intrahepatic Cholangiocarcinoma-PAICC Regulates Intrahepatic Cholangiocarcinoma Tumor Growth *In Vivo*

**Figure 5:**
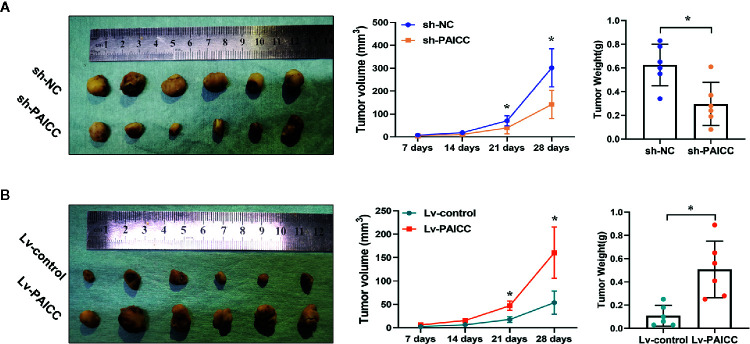
lncRNA-PAICC can regulate ICC growth *in vivo*. **(A)** Subcutaneous transplanted tumors in the nude mice were established using HUCCT-1 cells. The three figures in the upper panel represented xenograft tumors, quantitative analysis of tumor volume and weight in nude mice, respectively. (upper panel). **(B)** Subcutaneous transplanted tumors ware established using QBC-939 cells. The three figures in upper panel represented xenograft tumors, quantitative analysis of tumor volume and weight in nude mice, respectively. (lower panel). Data are mean ± SD (n = 6). Data between two groups were tested using independent sample t-test. *P < 0.05.

To investigate whether lncRNA-PAICC has an effect on ICC growth *in vivo*, we conducted a subcutaneous tumor formation experiment in nude mice. The *in vivo* results showed that the growth of tumors derived from xenografts lacking lncRNA-PAICC was significantly inhibited compared with tumors developed from mock-infected HUCCT-1 cells (**Figure 5A** upper panel). In contrast, the tumors derived from the control xenografts were significantly smaller than those in the QBC-939 cells with exogenous overexpression of lncRNA-PAICC (**Figure 5B** lower panel). In summary, these results suggested that lncRNA-PAICC promotes ICC cell invasion and tumor growth both *in vitro* and *in vivo*.

### Intrahepatic Cholangiocarcinoma-PAICC Exerts Its Competitive Endogenous RNA Function by Directly Sponging microRNAs

**Figure 6:**
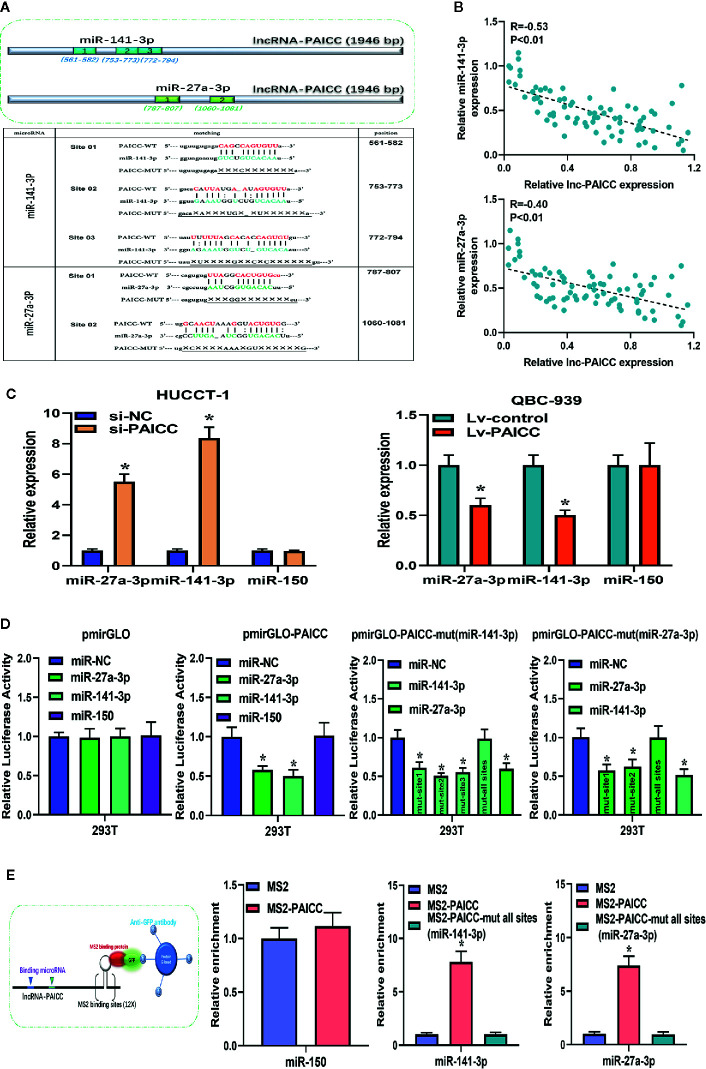
lncRNA-PAICC exerts its competitive endogenous RNA function by directly sponging microRNAs. **(A)** The top image shows the predicted binding sites for miR-141-3p and miR-27a-3p in lncRNA-PAICC. The following image shows miR-141-3p and miR-27a-3p binding sites predicted in the transcripts of lncRNA-PAICC. Target mutation underlined. **(B)** The correlation between lncRNA-PAICC transcript level with miR-141-3p (upper panel) and miR-27a-3p (low panel) transcript level was detected in 76 ICC tissues, respectively. The 2^−△△Ct^ values were subjected to Spearman’s correlation analysis. **(C)** These two microRNAs relative expression levels in HuCCT-1 and QBC-939 cells after transfection *via* RT–PCR. Data are mean ± SD. *P < 0.05. The experiment was repeated three times. **(D)** Luciferase activity in 293T cells co-transfected with miR-141-3p and miR-27a-3p and luciferase reporters that were empty or contained lncRNA-PAICC or the indicated mutant transcript. Data are mean ± SD. *P < 0.05. The experiment was repeated three times. **(E)** The schematic diagram of the MS2-RIP experiment is shown. After MS2-RIP, RT-PCR was used to detect miRNAs that can endogenously bind to lncRNA-PAICC, and the negative control was miR-150.

Currently, many lncRNAs and pseudogene RNAs have been hypothesized to be competitive endogenous RNAs (ceRNAs) or “RNA sponges” that interact with microRNAs and sequester these molecules to impair their regulation of target mRNAs (22). Thus, we investigated whether lncRNA-PAICC may function as a ceRNA. According to the results of our previous bioinformatics analysis, miR-141-3p and miR-27a-3p are likely to be sponged by lncRNA-PAICC. The binding schematic and sites of lncRNA-PAICC with these two microRNAs are shown in **Figure 6A** (upper panel). According to the assumption of ceRNA, lncRNA and microRNA should be negatively correlated at the expression level. Therefore, we further analyzed the correlation between the expression levels of lncRNA-PAICC and the two microRNAs in 76 human ICC specimens by RT-PCR. As shown in **Figure 6B**, Spearman’s correlation analysis suggested that the transcript level of lncRNA-PAICC was significantly negatively correlated with that of miR-141-3p (r = −0.53, p < 0.01; **Figure 6B**upper panel and miR-27a-3p (r = −0.40, p < 0.05; **Figure 6B**, lower panel). More importantly, RT-PCR showed that after lncRNA-PAICC was silenced, the expression of miR-141-3p and miR-27a-3p in HUCCT-1 cells significantly increased. After lncRNA-PAICC was overexpressed in QBC-939 cells, however, the expression of miR-141-3p and miR-27a-3p was dramatically reduced (**Figure 6C**). Using the full sequence of lncRNA-PAICC, we performed a luciferase reporter assay to verify the combination of these two miRNAs and lncRNA-PAICC in 293T cells. LncRNA-PAICC has three and two potential binding sites with miR-141-3p and miR-27a-3p, respectively, as shown in **Figure 6A** (lower panel). To verify the specificity and comprehensiveness of these binding sites, we designed luciferase reporter genes containing mutant lncRNA-PAICC sequences for each of the potential binding sites. **Figure 6D** shows that both miR-141-3p and miR-27a-3p reduced the luciferase activity of the lncRNA-PAICC reporter vector but did not reduce the luciferase activity of the empty vector. In addition, miR-150 did not reduce the luciferase activity. When the three potential binding sites of lncRNA-PAICC for miR-141-3p were mutated, the luciferase activity decreased to varying degrees. When all three potential sites in the lncRNA-PAICC sequence were mutated, miR-141-3p did not reduce the luciferase activity of the reporter vector. When both potential binding sites in the lncRNA-PAICC sequence were mutated, miR-27a-3p did not reduce the luciferase activity of the reporter vector.

To verify the binding of these two miRNAs to lncRNA-PAICC at the endogenous level, MS2-RNA immunoprecipitation (MS2-RIP) was performed to pull down the endogenous miRNAs related to lncRNA-PAICC. We engineered the following sequences: the empty vector (MS2); the vector containing the complete sequence of lncRNA-PAICC (MS2-PAICC); the vector targeting lncRNA-PAICC with all sites mutated in the miR-141-3p-binding site [designated PAICC-mut all sites (miR-141-3p)]; and the vector containing lncRNA-PAICC with all sites mutated in the miR-27a-3p-binding site [designated PAICC-mut all sites (miR-27a-3p)]. Subsequent RT-PCR results showed that compared with MS2 and the corresponding mutant vectors, lncRNA-PAICC-RIP was enriched for miR-141-3p and miR-27a-3p in HUCCT-1 cells (**Figure 6E**).

Thus, these results suggested that lncRNA-PAICC plays a regulatory role as a ceRNA through directly sponging miR-141-3p and miR-27a-3p.

### Intrahepatic Cholangiocarcinoma-PAICC Requires miR-141-3p and miR-27a-3p to Facilitate Proliferation, Facilitate Invasion, and Activate the Hippo Pathway in Intrahepatic Cholangiocarcinoma Cells

The GO annotation and KEGG pathway analyses (**Figures 1B, C**) indicated that many target genes are involved in a variety of cellular pathways, among which, the Hippo pathway is involved in the progress of ICC, and the expression of the main effector molecule, YAP1, in ICC is significantly elevated compared to corresponding para-tumor tissue. It is well known that YAP1 regulates cell proliferation and differentiation in a variety of cell lineages (23). In addition, we predicted through three online databases that YAP1 is the common downstream target gene of miR-141-3p and miR-27a-3p. The YAP1 3′UTR sequence containing miR-141-3p- and miR-27a-3p-binding sites is shown **in** **Supplementary Figure 3A**, and the complementary regions between these different species are also highly conserved.

To validate the role of miR-141-3p and miR-27a-3p in tumorigenesis by targeting YAP1, a luciferase reporter vector of the YAP1 3’UTR sequence containing the target sites of miR-141-3p and miR-27a-3p was constructed in 293T cells. The results showed that the luciferase activity of the YAP1 reporter gene vector was reduced after the miR-141-3p and miR-27a-3p mimics were transfected without affecting the luciferase activity of the mutant vector (**Supplementary Figure 3B**). After overexpression and knockdown of these two miRNAs (**Supplementary Figure 3C**), western blotting showed that miR-141-3p and miR-27a-3p reduced YAP1 protein levels, and it also showed that anti-miR-141-3p and anti-miR-27a-3p increased YAP1 protein levels (**Supplementary Figure 3D**). The above results indicated that YAP1 is a common downstream target gene of miR-141-3p and miR-27a-3p.

Based on the theory of ceRNA proposed by Pandolfi (24), lncRNA-PAICC may share the regulatory miRNAs with their targets. As expected, our previous data confirmed that lncRNA-PAICC regulated the expression level of YAP1 both at the mRNA and protein levels.

**Figure 7:**
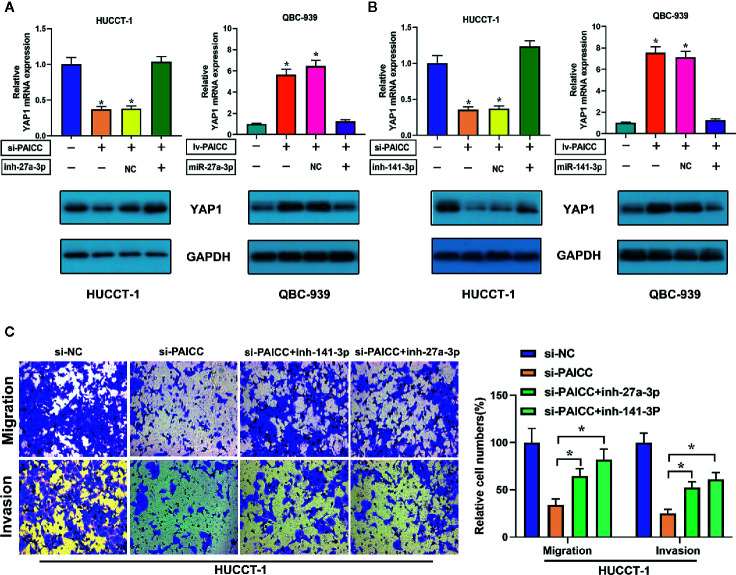
lncRNA-PAICC requires miR-141-3p and miR-27a-3p to promote proliferation and metastasis and activate the Hippo-YAP pathway in ICC cells. **(A)** YAP1 mRNA (upper panel) and corresponding protein (lower panel) levels in stable HUCCT-1 and QBC-939 cell lines after transfection of the indicated miR-27a-3p inhibitors or mimics. Data are mean ± SD. **(B)** YAP1 mRNA (upper panel) and corresponding protein (lower panel) levels in stable HUCCT-1 and QBC-939 cell lines after transfection of the indicated miR-141-3p inhibitors or mimics. Data are mean ± SD. **(C)** Transwell migration and invasion assays of lncRNA-PAICC-silenced HUCCT-1 cells treated with the indicated microRNA inhibitors. Data are mean ± SD. *P < 0.05. All the experiments were repeated three times.

In a corresponding rescue experiment, we inhibited the expression of miR-141-3p and miR-27a-3p in lncRNA-PAICC-silenced HUCCT-1 cells. The decreased expression of YAP1 was reversed after inhibition of miR-27a-3p and miR-141-3p in lncRNA-PAICC-silenced HUCCT-1 cells (**Figures 7A**, left panel; **B**, left panel). In contrast, the increase in YAP1 expression was abrogated after overexpression of miR-27a-3p and miR-141-3p in lncRNA-PAICC-overexpressing QBC-939 cells (**Figures 7A**, right panel; **B**, right panel). We also inhibited miR-141-3p and miR-27a-3p in lncRNA-PAICC-silenced HUCCT-1 cells, and we found that inhibition of these miRNAs partially offset the migration and invasion damage caused by lncRNA-PAICC knockdown in Transwell assays (**Figure 7C**).

**Figure 8:**
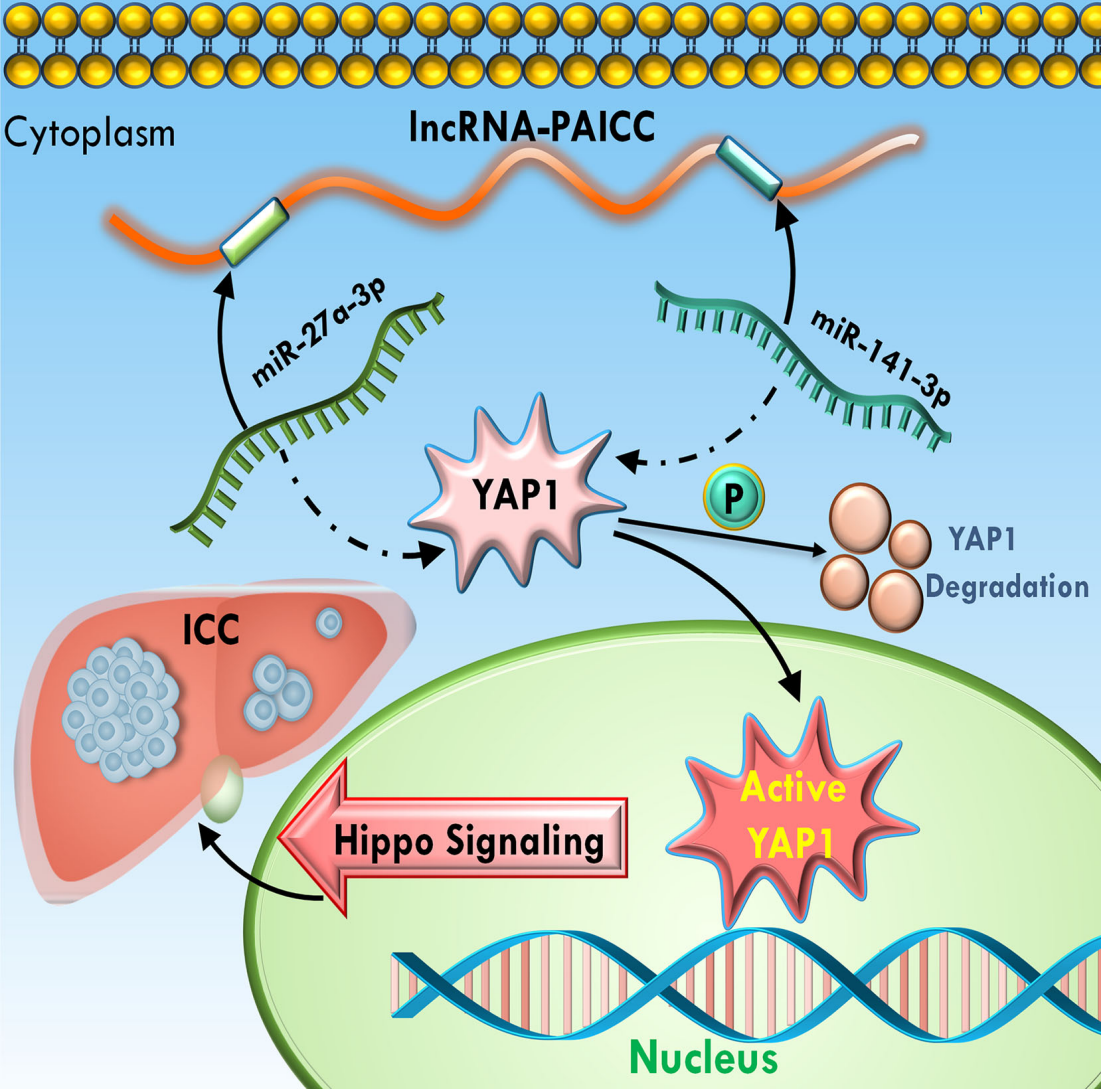
Schematic summary of the functions of lncRNA-PAICC in ICC cells. lncRNA-PAICC promotes ICC cell proliferation and tumor growth through the lncRNA-PAICC-miR-141-3p/27a-3p-Yap1 axis.

Thus, the above results suggested that lncRNA-PAICC plays a crucial role in regulating the expression of the YAP1 oncogene by competitively binding miR-141-3p and miR-27A-3p. More importantly, the interaction with miR-141-3P and miR-27a-3p is critical for lncRNA-PAICC to activate the Hippo pathway and thus promote the development of ICC (**Figure 8**).

## Discussion

Intrahepatic cholangiocarcinoma (ICC) is a highly heterogeneous devastating malignancy with a variety of causes and early metastasis and recurrence. Despite recent advances in ICC diagnostic standards and management, the prognosis for this highly lethal form of cancer remains poor (25, 26). A comprehensive and in-depth exploration of the underlying mechanisms of ICC tumorigenesis is beneficial to identify valuable diagnostic or therapeutic targets.

In this work, we identified that the expression of the novel lncRNA, lncRNA-PAICC, was upregulated in human ICC specimens through data mining. More importantly, we found that lncRNA-PAICC is a key regulator of YAP1 and facilitates the ICC proliferative invasion and metastasis cascade by multilevel *in vitro* and *in vivo* studies. Furthermore, the present study found that lncRNA-PAICC is significantly associated with advanced tumor stage and more unfavorable prognosis in patients with ICC. These results indicated that lncRNA-PAICC acts as an oncogenic lncRNA to promote oncogenesis in ICC.

The concept of competitive endogenous RNA (ceRNA) was first proposed by Pandolfi. CeRNA is considered a new type of gene expression regulation mode, that is, transcripts such as mRNAs, pseudogene and lncRNAs compete with the same microRNA through microRNA response elements (MREs), thereby diluting the concentration of free microRNAs in the cell and reducing the inhibition of microRNAs on mRNAs, in turn, increasing the expression of target genes to regulate cell function and phenotype (24). This model of ceRNA regulation has been shown to play a key role in tumor formation and development. LncRNA-ATB has been reported to be an endogenous “sponge” that upregulates ZEB1 and ZEB2 by competitively binding to the miR-200 family, thereby inducing EMT and liver cancer invasion (27). LncRNA SNHG7 sponges miR-216b to promote proliferation and liver metastasis of colorectal cancer through upregulating GALNT1 (16). Similarly, lncRNA-LINC01133 promotes the evolution of gallbladder tumor by sponging miRNAs (14). In our study, we sought to investigate whether lncRNA-PAICC functions as a ceRNA in ICC. First, we identified the target binding sites of miR-141-3p and miR-27a-3p in lncRNA-PAICC by bioinformatics software, and we further confirmed the binding sites using a dual luciferase reporter assay and a MS2-RIP experiment. The results demonstrated that lncRNA-PAICC competed with these two miRNAs. We also confirmed that lncRNA-PAICC competitively combined these two miRNAs and downregulated their expression levels in ICC cells. Furthermore, in human ICC samples, miR-141-3p and miR-27a-3p were negatively correlated with lncRNA-PAICC expression. Based on these results, we hypothesized that miR-141-3p and miR-27a-3p are required for at least part of the carcinogenesis of lncRNA-PAICC. To confirm this hypothesis, we designed rescue experiments and ectopically expressed lncRNA-PAICC mutations. As expected, this competition was essential for lncRNA-PAICC to display its regulatory function in facilitating the ICC progress.

Relevant studies have shown that the Hippo pathway regulates the size of organs by regulating cell proliferation, apoptosis and stem cell self-renewal in both drosophila and mammals. Anomalies in this pathway can lead to excessive tissue growth. In addition, many studies have confirmed that the Hippo pathway plays an important role in cancer occurrence, tissue regeneration and the regulation of stem cell functions (6, 28, 29). Currently, several studies have investigated the role of the Hippo pathway in various malignancies, including the potential involvement of YAP1 (30–32). However, the role and mechanism of YAP1 in intrahepatic cholangiocarcinoma remains unclear. In this research, our focus was to explore the role of the Hippo pathway in ICC mediated by the molecular sponge effect of lncRNA. Therefore, our hypothesis suggested that YAP1 is a downstream target gene of the ceRNA regulatory axis. Importantly, YAP1, as a direct downstream effector of the Hippo pathway, has been confirmed to exist as an oncogene with increased expression in a variety of tumors (30, 33). Bioinformatics prediction and dual luciferase reporter assays further confirmed that YAP1 is a common downstream target gene of miR-141-3p and miR-27a-3p, which are positively regulated by lncRNA-PAICC. As expected, our experiments indicated that this positive relationship depended, at least in part, on the competitive effects of both miRNAs. The present study indicated that lncRNA-lncRNA-PAICC activates the Hippo pathway in ICC cells by relying on miR-141-3p and miR-27a-3p.

Although these studies have revealed important findings, they also have limitations. First, we still lack a comprehensive understanding of the regulation of a single lncRNA because the mechanism of ceRNA is only one of its many modes of action (11, 34). Most tumors occur due to the cumulative mutation of multiple genes and complex processes involving many signal transduction pathways (35, 36). Second, further research on the Hippo pathway is required to understand its interaction with other signaling pathways (37–39). We also need to further explore other mechanisms, such as evolution, drug resistance and targeted metastasis, for the heterogeneous and highly aggressive tumor.

Through mining of a large database, we discovered and identified lncRNA-PAICC as a marker of poor prognosis that plays an important role as a carcinogenic lncRNA in ICC. Importantly, lncRNA-PAICC attenuates miR-141-3p- and miR-27a-3p-dependent downregulation of YAP1 by acting as a molecular sponge, thereby promoting tumorigenesis of ICC cells and activating the Hippo pathway. Our results suggested a new perspective and insight for further elucidating the molecular pathogenesis of ICC, and they presented a potential new target for future ICC therapies.

## Data Availability Statement

The original contributions presented in the study are included in the article/**Supplementary Material**; further inquiries can be directed to the corresponding authors.

## Ethics Statement

The studies involving human participants were reviewed and approved by the ethics committee of the Third Affiliated Hospital of Sun Yat-sen University. The patients/participants provided their written informed consent to participate in this study. The animal study was reviewed and approved by the committee of Sun Yat-sen University, Guangzhou, China. Written informed consent was obtained from the individuals for the publication of any potentially identifiable images or data included in this article.

## Author Contributions

HL and GC conceived and directed this research. XC, LZ, SZ, and QY contributed to the project design. LX, XC, JY, and LZ conducted the experiments. QY, YH, and LX analyzed the bioinformatics data. LZ, XC, SZ, and JY helped provide samples, data, and comments on the manuscript. LZ, XC, and QY helped analyze and interpret the data. Reagents, materials, and contributions were contributed by LZ, XC, SZ, JY, and QY. LX, XC, and JY were responsible for drafting the manuscript. All authors contributed to the article and approved the submitted version.

## Funding

This study was supported by the National Science Foundation of China (Grant No. 81172038, 82070673, 81870447, 81170422 and 82002587), the Scientific and Technological Projects of Guangdong Province (2017A020215178, 2019B020236003 and 2020B1212060019) and China Postdoctoral Science Foundation (2020TQ0370).

## Conflict of Interest

Author YH was employed by the company Forevergen Biosciences Co., Ltd.

The remaining authors declare that the research was conducted in the absence of any commercial or financial Relationships that could be construed as a potential conflict of interest.

## References

[1] Bridgewater J Galle PR Khan SA Llovet JM Park JW Patel T Guidelines for the diagnosis and management of intrahepatic cholangiocarcinoma. J Hepatol (2014) 60:1268–89. 10.1016/j.jhep.2014.01.021 24681130

[2] Weber SM Ribero D O’Reilly EM Kokudo N Miyazaki M Pawlik TM Intrahepatic cholangiocarcinoma: expert consensus statement. HPB (Oxford) (2015) 17:669–80. 10.1111/hpb.12441 PMC4527852 26172134

[3] Kelley RK Bridgewater J Gores GJ Zhu AX Systemic therapies for intrahepatic cholangiocarcinoma. J Hepatol (2020) 72:353–63. 10.1016/j.jhep.2019.10.009 31954497

[4] Rizvi S Gores GJ Emerging molecular therapeutic targets for cholangiocarcinoma. J Hepatol (2017) 67:632–44. 10.1016/j.jhep.2017.03.026 PMC5563275 28389139

[5] Blechacz B Gores GJ Tumor-specific marker genes for intrahepatic cholangiocarcinoma: utility and mechanistic insight. J Hepatol (2008) 49:160–2. 10.1016/j.jhep.2008.05.001 PMC2885696 18538440

[6] Badouel C McNeill H SnapShot: The hippo signaling pathway. Cell (2011) 145:484–84 e1. 10.1016/j.cell.2011.04.009 21529719

[7] Ma Y Yang Y Wang F Wei Q Qin H Hippo-YAP signaling pathway: A new paradigm for cancer therapy. Int J Cancer (2015) 137:2275–86. 10.1002/ijc.29073 25042563

[8] Zou S Li J Zhou H Frech C Jiang X Chu JS Mutational landscape of intrahepatic cholangiocarcinoma. Nat Commun (2014) 5:5696. 10.1038/ncomms6696 25526346

[9] Moeini A Sia D Bardeesy N Mazzaferro V Llovet JM Molecular Pathogenesis and Targeted Therapies for Intrahepatic Cholangiocarcinoma. Clin Cancer Res (2016) 22:291–300. 10.1158/1078-0432.CCR-14-3296 26405193 PMC12224570

[10] Chen L Yan HX Yang W Hu L Yu LX Liu Q The role of microRNA expression pattern in human intrahepatic cholangiocarcinoma. J Hepatol (2009) 50:358–69. 10.1016/j.jhep.2008.09.015 19070389

[11] Schmitt AM Chang HY Long Noncoding RNAs in Cancer Pathways. Cancer Cell (2016) 29:452–63. 10.1016/j.ccell.2016.03.010 PMC4831138 27070700

[12] Wright MW A short guide to long non-coding RNA gene nomenclature. Hum Genomics (2014) 8:7. 10.1186/1479-7364-8-7 24716852 PMC4021045

[13] Abbastabar M Sarfi M Golestani A Khalili E lncRNA involvement in hepatocellular carcinoma metastasis and prognosis. EXCLI J (2018) 17:900–13. 10.17179/excli2018-1541 PMC6295623 30564069

[14] Wu XS Wang F Li HF Hu YP Jiang L Zhang F LncRNA-PAGBC acts as a microRNA sponge and promotes gallbladder tumorigenesis. EMBO Rep (2017) 18:1837–53. 10.15252/embr.201744147 PMC5623869 28887321

[15] Xu Y Yao Y Jiang X Zhong X Wang Z Li C SP1-induced upregulation of lncRNA SPRY4-IT1 exerts oncogenic properties by scaffolding EZH2/LSD1/DNMT1 and sponging miR-101-3p in cholangiocarcinoma. J Exp Clin Cancer Res (2018) 37:81. 10.1186/s13046-018-0747-x 29642935 PMC5896100

[16] Shan Y Ma J Pan Y Hu J Liu B Jia L LncRNA SNHG7 sponges miR-216b to promote proliferation and liver metastasis of colorectal cancer through upregulating GALNT1. Cell Death Dis (2018) 9:722. 10.1038/s41419-018-0759-7 29915311 PMC6006356

[17] Li S Zhao B Zhao H Shang C Zhang M Xiong X Silencing of Long Non-coding RNA SMAD5-AS1 Reverses Epithelial Mesenchymal Transition in Nasopharyngeal Carcinoma via microRNA-195-Dependent Inhibition of SMAD5. Front Oncol (2019) 9:1246. 10.3389/fonc.2019.01246 31921616 PMC6923203

[18] Liu H Deng H Zhao Y Li C Liang Y LncRNA XIST/miR-34a axis modulates the cell proliferation and tumor growth of thyroid cancer through MET-PI3K-AKT signaling. J Exp Clin Cancer Res (2018) 37:279. 10.1186/s13046-018-0950-9 30463570 PMC6249781

[19] Ren X Chen C Luo Y Liu M Li Y Zheng S lncRNA-PLACT1 sustains activation of NF-kappaB pathway through a positive feedback loop with IkappaBalpha/E2F1 axis in pancreatic cancer. Mol Cancer (2020) 19:35. 10.1186/s12943-020-01153-1 32085715 PMC7033942

[20] Wei L Wang X Lv L Liu J Xing H Song Y The emerging role of microRNAs and long noncoding RNAs in drug resistance of hepatocellular carcinoma. Mol Cancer (2019) 18:147. 10.1186/s12943-019-1086-z 31651347 PMC6814027

[21] Wang L Park HJ Dasari S Wang S Kocher J-P Li W CPAT: Coding-Potential Assessment Tool using an alignment-free logistic regression model. Nucleic Acids Res (2013) 41:e74–4. 10.1093/nar/gkt006 PMC3616698 23335781

[22] Zhang S Xiao J Chai Y Du YY Liu Z Huang K LncRNA-CCAT1 Promotes Migration, Invasion, and EMT in Intrahepatic Cholangiocarcinoma Through Suppressing miR-152. Dig Dis Sci (2017) 62:3050–58. 10.1007/s10620-017-4759-8 28921383

[23] Maugeri-Sacca M De Maria R The Hippo pathway in normal development and cancer. Pharmacol Ther (2018) 186:60–72. 10.1016/j.pharmthera.2017.12.011 29305295

[24] Salmena L Poliseno L Tay Y Kats L Pandolfi PP A ceRNA hypothesis: the Rosetta Stone of a hidden RNA language? Cell (2011) 146:353–8. 10.1016/j.cell.2011.07.014 PMC3235919 21802130

[25] Hoyos S Navas MC Restrepo JC Botero RC Current controversies in cholangiocarcinoma. Biochim Biophys Acta Mol Basis Dis (2018) 1864:1461–67. 10.1016/j.bbadis.2017.07.027 28756216

[26] Sirica AE Gores GJ Groopman JD Selaru FM Strazzabosco M Wei Wang X Intrahepatic Cholangiocarcinoma: Continuing Challenges and Translational Advances. Hepatology (2019) 69:1803–15. 10.1002/hep.30289 PMC6433548 30251463

[27] Yuan JH Yang F Wang F Ma JZ Guo YJ Tao QF A long noncoding RNA activated by TGF-beta promotes the invasion-metastasis cascade in hepatocellular carcinoma. Cancer Cell (2014) 25:666–81. 10.1016/j.ccr.2014.03.010 24768205

[28] Patel SH Camargo FD Yimlamai D Hippo Signaling in the Liver Regulates Organ Size, Cell Fate, and Carcinogenesis. Gastroenterology (2017) 152:533–45. 10.1053/j.gastro.2016.10.047 PMC5285449 28003097

[29] Shi X Zhu HR Liu TT Shen XZ Zhu JM The Hippo pathway in hepatocellular carcinoma: Non-coding RNAs in action. Cancer Lett (2017) 400:175–82. 10.1016/j.canlet.2017.04.032 28461246

[30] Wang J Huang F Shi Y Zhang Q Xu S Yao Y RP11-323N12.5 promotes the malignancy and immunosuppression of human gastric cancer by increasing YAP1 transcription. Gastric Cancer (2020). 10.1007/s10120-020-01099-9 32623586

[31] Li H Wolfe A Septer S Edwards G Zhong X Abdulkarim AB Deregulation of Hippo kinase signalling in human hepatic malignancies. Liver Int (2012) 32:38–47. 10.1111/j.1478-3231.2011.02646.x 22098159 PMC4175712

[32] Kim W Khan SK Gvozdenovic-Jeremic J Kim Y Dahlman J Kim H Hippo signaling interactions with Wnt/beta-catenin and Notch signaling repress liver tumorigenesis. J Clin Invest (2017) 127:137–52. 10.1172/JCI88486 PMC5199712 27869648

[33] Bai H Gayyed MF Lam-Himlin DM Klein AP Nayar SK Xu Y Expression of Yes-associated protein modulates Survivin expression in primary liver malignancies. Hum Pathol (2012) 43:1376–85. 10.1016/j.humpath.2011.12.001 PMC3384990 22436626

[34] Bekric D Neureiter D Ritter M Jakab M Gaisberger M Pichler M Long Non-Coding RNAs in Biliary Tract Cancer-An Up-to-Date Review. J Clin Med (2020) 9:1200–31. 10.3390/jcm9041200 PMC7231154 32331331

[35] Nepal C O’Rourke CJ Oliveira D Taranta A Shema S Gautam P Genomic perturbations reveal distinct regulatory networks in intrahepatic cholangiocarcinoma. Hepatology (2018) 68:949–63. 10.1002/hep.29764 PMC6599967 29278425

[36] Haga H Patel T Molecular diagnosis of intrahepatic cholangiocarcinoma. J Hepatobiliary Pancreat Sci (2015) 22:114–23. 10.1002/jhbp.156 PMC4427040 25267595

[37] Sugihara T Isomoto H Gores G Smoot R YAP and the Hippo pathway in cholangiocarcinoma. J Gastroenterol (2019) 54:485–91. 10.1007/s00535-019-01563-z PMC6536462 30815737

[38] Sugimachi K Nishio M Aishima S Kuroda Y Iguchi T Komatsu H Altered Expression of Hippo Signaling Pathway Molecules in Intrahepatic Cholangiocarcinoma. Oncology (2017) 93:67–74. 10.1159/000463390 28448997

[39] Marti P Stein C Blumer T Abraham Y Dill MT Pikiolek M YAP promotes proliferation, chemoresistance, and angiogenesis in human cholangiocarcinoma through TEAD transcription factors. Hepatology (2015) 62:1497–510. 10.1002/hep.27992 26173433

