# Supplementary material for: Long Non-Coding RNA-PAICC Promotes the Tumorigenesis of Human Intrahepatic Cholangiocarcinoma by Increasing YAP1 Transcription
Source: Front Oncol. 2021 Jan 8;10:595533. doi: 10.3389/fonc.2020.595533 (PMC7856545; doi:10.3389/fonc.2020.595533)
Supplement: Supplementary file 1 [file DataSheet_1.docx]

**Supplementary Materials and Methods**

**Cell Cultures and Treatments**

Human intrahepatic cholangiocarcinoma derived cell lines QBC-939, HUCCT-1 and HCCC-9810, and a normal human intrahepatic bile duct epithelial cell（HIBEC）were obtained from Shanghai Institute for Biological Science(Shanghai, China). All cells were grown in DMEM medium containing 10% FBS (Gibco)and cultured in 5% CO_2_ at 37°C.

**Reverse Transcription Reactions and Quantitative real-time PCR**

The total RNA was isolated using Trizol reagent (Invitrogen). Subsequently, RNA was reversely transcribed into cDNA (ES science). SYBR-Green II PCR kit (RiboBio) was used to detect the level of miRNA by RT-PCR, U6 snRNA was used as a reference. SYBR Green PCR kit (ES science) was used to quantify the expression levels of lncRNA and mRNA, and GAPDH was used as a reference. Each reaction is repeated three times. The relative expression levels were calculated by 2^-△△Ct^ method and converted into fold change. Primer sequences are shown in Table S2 in appendix.

**Isolation of Cytoplasmic and Nuclear RNA**

To determine the cellular localization of the lncRNA-PAICC, nuclear fragments were isolated from the cytoplasm according to instructions from Paris Kit (Life Technologies, Thermo Fisher Scientific). RNA was extracted from cytoplasm and nucleus respectively. RT-PCR was used to detect the expression ratio of specific RNA molecules between cytoplasm and nuclear fragments. β-actin and U6 act as cytoplasmic and nuclear controls, respectively.

**Fluorescence in Situ Hybridization**

A digoxin -labelled (Roche, Mannheim, Germany) lncRNA-PAICC probe was synthesized in vitro (Appendix Table S1), and a negative control was used for fluorescence in situ hybridization (FISH). Cells were seeded onto autoclaved glass slides. The following procedures were performed as previously described (21).

**Cell Viability Analyses and Colony Formation Assay**

Cell viability was examined according to the manufacturer’s instructions with cell-counting kit-8 (Yiyuan Biotechnologies). Cells were inoculated into 96-well plates (5000 cells/well in 100 μL of medium) and 10 μL of CCK-8 solution was added to each hole at the same time every day, the absorbance (450 nm) of each hole was measured after incubation for 2 h at 37 °C. **For colony formation assay,** cells were incubated for 2 weeks in a 6-well plate with a density of 2000 cells/well. The colonies were fixed with 75% alcohol and stained with crystal violet. Count the colony ≥50 cells under the microscope.

**Wound Healing Assay**

The cells were seeded in a 6-well plate and cultured overnight. Scratch the cell monolayer with the tip of a plastic 10-leds l microtube, then wash it twice with PBS and add fresh growth medium to the each well. Subsequently, the cells were transfected for 48 hours as described above. Images of different stages of wound healing were taken through a microscope at 0 hours and 24 hours. Use Image-Pro Plus to quantify relative cell viability.

**Migration and Invasion Assay**

A transwell chamber (Corning, New York) was used to determine the migration capabilities of cells. The cells (10×10^4^) suspended in serum-free medium were transferred to the upper chamber, and a medium containing 10% fetal bovine serum was added to the lower chamber as a chemokine. After 24 hours, the cells that migrated to the lower surface of the membrane were fixed with 75% methanol and stained with crystal violet. Under microscope, the migration ability was evaluated by counting cells, and 5 random fields were analyzed in each chamber.

For invasion assays, chamber inserts were pre-coated with 50 μL 1:9 mixture of BD Matrigel (BD Biosciences) and DMEM for overnight under sterile conditions. Then 2×10^5^ cells were seeded in the upper chamber. After 48 h, cells on the top side of each insert were scraped off gently, and then fixed in methanol, and stained by crystal violet.

**Vectors Construction**

The cDNA encoding lncRNA-PAICC was synthesized by Genechem (Shanghai, China), and subcloned to the NheI and XhoI loci of PCR-MS2-12X vector (Addgene), which was named pSL-MS2-PAICC. For lncRNA-PAICC mutants, ten nucleotides corresponding to the lncRNA-PAICC of miR-141-3p and miR-27a-3p 5 'UTR were deleted at least from the Mut constructs, and were named as pSL-MS2-PAICC-mut(miR-141-3p) and pSL-MS2-PAICC-mut(miR-27a-3p), respectively**.** For the luciferase reporter experiment, we first double digested the NheI and XhoI, the sites of pSL-MS2-PAICC-mut (miR-141-3p) or pSL-PAICC-mut (miR-27a-3p) XhoI, and then subcloned the fragments containing lncRNA-PAICC into the pmirGLO vector (Promega, Madison, WI), and then named them pmirGLO-PAICC pmirGLO-PAICC-mut (miR-141-3p) or pmirGLO-PAICC-mut (miR-27a-3p), respectively. The 3' untranslated region (3'-UTR) of YAP1 containing miR-141-3p and miR-27a-3p recognition sequences was amplified by PCR and subcloned into MluI and HindIII loci of the pMIR-Report Fluc vector (Ambion). Six nucleotides in the 3'-UTR corresponding to the 5'-UTR of miR-141-3p and miR-27a-3p were missing at least in the Mut constructs. All the constructs were confirmed by DNA sequencing.

**Generation of Stable ICC Cell Lines**

To obtain cell lines stably expressing lncRNA-PAICC, lncRNA-PAICC cDNA was PCR-amplified and subcloned to the lentiviral vector Ubi-MCS-SV40-EGFP-IRES-puromycin (Genechem). Recombinant lentiviruses containing the lncRNA-PAICC gene (Lv-PAICC) were produced by GeneChem. QBC-939 were infected with concentrated virus and were selected with 1 ug/mL puromycin for 2 weeks. Real-time PCR was performed to identify the stably overexpressing cell lines. For the construction of cell lines stably expressing lncRNA-PAICC shRNA, the shRNA sequences were shown in Appendix Table S1. shRNA-lncRNA-PAICC and negative control shRNA were synthesized and inserted into hU6-MCS-Ubiquitin-EGFP-IRES-puromycin lentiviral vector. Recombinant lentiviruses expressing lncRNA-PAICC-shRNA or negative control shRNA (Lv-sh PAICC and Lv-sh NC, respectively) were produced by Genechem. HUCCT-1 cell was infected with concentrated virus, and the culture medium was replaced after 24-h incubation. Then, cells were treated with 2 ug/ml puromycin for 2 weeks for the selection of stable cell lines. The expression of lncRNA-PAICC in the stable cell lines was validated by RT-PCR analysis.

**Transient Transfection**

Transfections were performed using the Lipofectamine 3000 kit (Invitrogen) according to the manufacturer’s instructions. The microRNA inhibitors, double-stranded microRNA mimics and their respective negative control RNAs (GenePharma) were introduced into cells at a final concentration of 50 nM. The cells were harvested at 48 h after transfection.

**Dual-luciferase Reporter Assay**

The pmirGLO, pmirGLO-PAICC or pmirGLO-PAICC-mut (miR-141-3p/miR-27a-3p) was co-transfected with miR-141-3p, the miR-27a-3p mimic or control in 293T cells. Relative luciferase activity was normalized to Renilla luciferase activity 24 h after transfection and was measured according to the manufacturer’s protocol using a dual-Luciferase Reporter Assay System (Promega, Wisconsin).

**MS2-RIP**

HUCCT-1 cells were co-transfected with pSL-MS2, pSL-MS2-PAICC, pSL-MS2-mut (miR-141-3p / 27a-3p) and pMS2-GFP (Addgene). The cells were used for RNA immunoprecipitation (RIP) experiments after 48 hours, as previously described above. RT-PCR was used to analyze the RNA fragments obtained by RIP.

**Western Blot Analysis**

Equivalent amounts of proteins were separated by SDS-PAGE and then transferred to polyvinylidene fluoride membranes (PVDF, Beyotime). The membrane was blocked with 5% nonfat milk. After that, the membrane was incubated with the corresponding primary antibody, including: YAP1 (1:1000, #14074, Cell Signaling Technology) and GAPDH (1:5000, #5174, Cell Signaling Technology), then washed with PBST, incubated with HRP-conjugated secondary antibodies for 2- h at room temperature. Detection was performed by ECL.

**Immunohistochemical**

Immunohistochemical experiments were performed on paraffin sections of ICC patients to observe the expression of YAP1. Slides were incubated at 4 °C overnight with primary anti-YAP1 (1;500, #14074, Cell Signaling Technology).

**Animal Studies**

Animal research was approved by the animal care and use committee of Sun Yat-sen University, Guangzhou, China. BALB/c nude mice (4-5 weeks old) for animal studies were purchased from Beijing Vital River Laboratory Animal Technology Co., Ltd., China. ICC cells were injected subcutaneously in the right flank of the nude mice. Tumor volume should be measured regularly (every 1 week) during the period of tumor-bearing nude mice. Four weeks after ICC cells inoculation, the tumors were collected.

**REFERENCES**

21. Khaitan D, Dinger M E, Mazar J, Crawford J, Smith M A, Mattick J S et al. The melanoma-upregulated long noncoding RNA SPRY4-IT1 modulates apoptosis and invasion. *Cancer Res*. (2011) 71:3852-62. doi: 10.1158/0008-5472.CAN-10-4460
