# Supplementary material for: Long Non-Coding RNA-PAICC Promotes the Tumorigenesis of Human Intrahepatic Cholangiocarcinoma by Increasing YAP1 Transcription
Source: Front Oncol. 2021 Jan 8;10:595533. doi: 10.3389/fonc.2020.595533 (PMC7856545; doi:10.3389/fonc.2020.595533)
Supplement: Supplementary file 2 [file Image_1.pdf]

A

Transcript: lnc-AMOTL2-1:1

Basic information

LNCipedia transcript ID: lnc-AMOTL2-1:1
LNCipedia gene ID: lnc-AMOTL2-1
Ensembl Gene ID: ENSG00000260633
Ensembl Transcript ID: ENST00000568384
Location (hg38): chr3:134347288-134349233
Strand: -
Class: intergenic
Sequence Ontology term: lincRNA
Transcript size: 1946 bp
Exons: 1
Sources: Gencode v13; Ensembl release 68 - Jul 2012; NONCODE v4; Ensembl release 75 - Feb 2014; Ensembl release 83 - Dec 2015; Ensembl release 87 - Dec 2016; Ensembl release 90 - Aug 2017; Ensembl release 92 - Apr 2018
Alternative transcript names: ENST00000568384.1; RP11-375I20.6-001; OTTHUMT00000431114.1; NONHSAT092234
Alternative gene names: ENSG00000260633.1; RP11-375I20.6; OTTHUMG00000175807.1; AC010207.1

B

This is the Table View of CPC results. Your task ID is 70DBE280-C5C1-11EA-A830-BA63F6185444 . You may use the ID to retrieve your results later in our view retrieve page . You can also view Raw Data of the CPC results.

ID	C/NC	CODING POTENTIAL SCORE	EVIDENCE	UTR-DB HITs	RNA-DB HITs
user_seq	noncoding	-1.18878	detail	search	search

EVIDENCE FEATURES SUMMARY

HOMOLOGY FEATURES	HIT NUM	0
	HIT SCORE	0.0
	FRAME SCORE	0.0
ORF_FRAMEFINDER	COVERAGE	10.48 %
	LOG-ODDS SCORE	31.53
	TYPE	Full

legend: non-coding … coding

C

Coding Potential Assessment Tool

Result for species name : hg19 with job ID :1568173729

Data ID	Sequence Name	RNA Size	ORF Size	Ficket Score	Hexamer Score	Coding Probability	Coding Label
0	RP11-375I20.6	1946	306	0.456	-0.0612531	0.0202654	no

D

```
0001 CACCAAACAC ACAGGCATGA AATACACTCA TGGGCTTGTC CACATGCACA CCTGCCACAA ACACCCATCA TAAGCCTCCA GGTAAACAAT ATGGGCATGC
0101 GCACTTATAC ACACACTTGC ACACACATAC ACATTTTTGG AGGCTTGAAT GCACACCCAC GCCCAACACA CACCCCCTCA GGCAGGCACA CATGGGTCCC
0201 CTAAACATGG ACTCAGTCTT CCTACAGGAG AATTTCTATT CTGAGCAAAC ACCCAGCATC TGCAGTCAAT GGCAAACAGA TGAATGGGAC AAAATTCACT
0301 CATGTGTCCT GGTGGCCCTA CCAATACAGC CCTGAAGGAG CAGGCAGCAG GCTTATCCCT TCACCAGCAG GCCTTCAGAA GGGCAAGACC AGGGTGGGGA
0401 TCAGGTTTGG CCAGGAGAAC ACTCAGAGCT TCTGGGTACT GACTCAAAGC CAGGGGCCGA CAGCCTTGTG CATTCCTGAC AAGTAGGTCA CCATGACAAG
0501 GAGCACTGGC TCACATGCTG GGACCTACGG TGACTGGAAA AGACTTGGGG AAAATCCAGC TGTTGTGAGA CAGCCAGTGT TAGTAGGTGC TGCCTTCCCA
0601 TACCAGTGAG TTAGGTTGGG TCTATGCAGC ACCGGAGCTA TGGAGTGTGT GTCAGCGGCA AGGGCTCCTG CTCAGCACAG CAAAGACTAG GAGAAGAAGA
0701 GGGCAGTGGC TTACCAGGGA CTGTAGCGCT GGGAAGGACG GGTGAGGCAG GGGACACATT ATGAATAGTG TTATTTTTTA GCACACCAGT GTGTTAGGCA
0801 CTGTGCTGGG TGTTTTTCAT GTATTATCTT GCAAGATACA GCCCTGCAAG TTAGATTTTA TTGCCTCCAT TTTACAGATG AGGAGACTGA GGCTCAGAGG
0901 GCTTAAATAA ATTACTCTAG GTCTTCAGCT AATAGGGGCA GAGCTCAGAT ATGAGTCAAG GCTTGTCTGA CTCCAGATCT CAGACTTTCT CAACATCTTC
1001 ATGTTGAAGC TAAAACTTGC ATTTTAAACT ATCAAAAGCA AGAGGGATCT AAGGAGACAT GGCAACTAAA GGTACTGTGG TATCCCGGAT GGGATCCTGG
1101 GACAGAAAAT AAACATGAAC TTTAGTTAAT GACAATTTTA ATTGTGATAC ATGTACTTTA CTAATGCAAA AGTGTTAATA ATAGAGGAAA CTGGGTGCAG
1201 GGTATACAGG AACTATTTAT ACTATCTTAA TGATTCTTCA AATCTAAAAC TGTCCTAAAA TAAAAGACTT ATTTTTTAAA CTTTGCATTT TTGCCAGCAG
1301 CAGGAAGCAG TAGATAACCA ACATTCCAAG GTGCAGCCTG TGTATAAGGC CTGAAGAGGC CAGGGCCCCT GGAAAGTTTC TCAAAATAAG AGGCCATTCT
1401 GGACTTTGTA CCACTGAGTT CCCATCACCT ACATAACTTG AAATAGAGAC ATTAATTGCA GGGCTGTGAT CACAGCCAAC ACCACCTTCC TAAATGGGGC
1501 AAAAGGGCGC TGAAGTCGGG CAGAGCAGCT GCAAACTGGC ATGAATTCCC AAGGCTGTGA CAGAGAATTC TTGCTGGGTG TGCCTGTACC TCCCTGACAA
1601 TGTGGCCTGG CCAAGCTGAC GTCCCTCCAT TTTCTCAACT GTTGCCACTA GAATGTAGTA GGAACAATGT ATAAGTCTTA TACTTCTTTT ATTAAATTTA
1701 TTCTTAGATA TCTTATTCTT AGTAATACTA CTATAAATGT AGTTGCTTTC TTGATTTCAT TTTCTGATAT TTTATTTCTA GTACATGAAA ATAAAATTCA
1801 TTTTTGTATA TTGATCTTAT GTTTTGTCAC CTTGCTGAGC TTGCTTATCA GTCCTAACAG TTTTTTAGTG GATTCCTTAG GATTTTCTAT ATACTGCAAG
1901 ATTATGGCAT CTACAAATAA AGATAGTTTA AGTTCTTTCT TTCCAA
```

Supplementary Figure 1 Basic information about lncRNA-PAICC comes from several online database.

(A) The basic annotation information for lncRNA-PAICC from LNCipedia database. **(B)** Coding ability prediction results of lncRNA-PAICC from Coding Potential Caculator. **(C)** Coding ability prediction results of lncRNA-PAICC from Coding-Potential Assessment Tool. **(D)** The full sequence information of lncRNA-PAICC (1946bp).
