# Supplementary material for: Long Non-Coding RNA-PAICC Promotes the Tumorigenesis of Human Intrahepatic Cholangiocarcinoma by Increasing YAP1 Transcription
Source: Front Oncol. 2021 Jan 8;10:595533. doi: 10.3389/fonc.2020.595533 (PMC7856545; doi:10.3389/fonc.2020.595533)
Supplement: Supplementary file 3 [file Image_2.pdf]

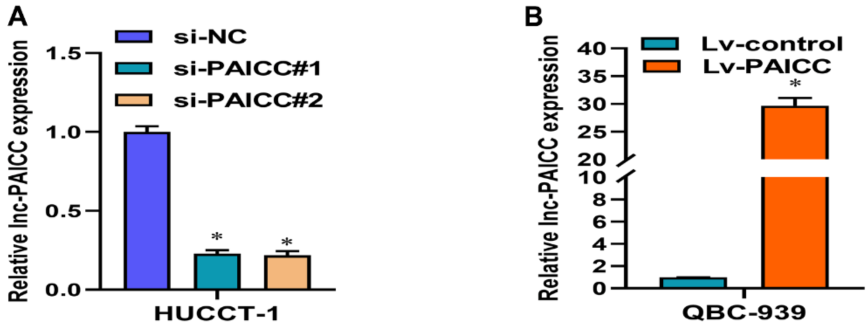

Supplementary Figure 2 The expression level verification of lncRNA-PAICC.
(A) After lncRNA-PAICC was silenced, its expression level was verified by RT-PCR. Data are mean ± SD. *P < 0.05. The experiment was repeated three times. **(B)** After lncRNA-PAICC was overexpressed, its expression level was verified by RT-PCR. Data are mean ± SD. *P < 0.05. The experiment was repeated three times.
