# Supplementary material for: Long Non-Coding RNA-PAICC Promotes the Tumorigenesis of Human Intrahepatic Cholangiocarcinoma by Increasing YAP1 Transcription
Source: Front Oncol. 2021 Jan 8;10:595533. doi: 10.3389/fonc.2020.595533 (PMC7856545; doi:10.3389/fonc.2020.595533)
Supplement: Supplementary file 4 [file Image_3.pdf]

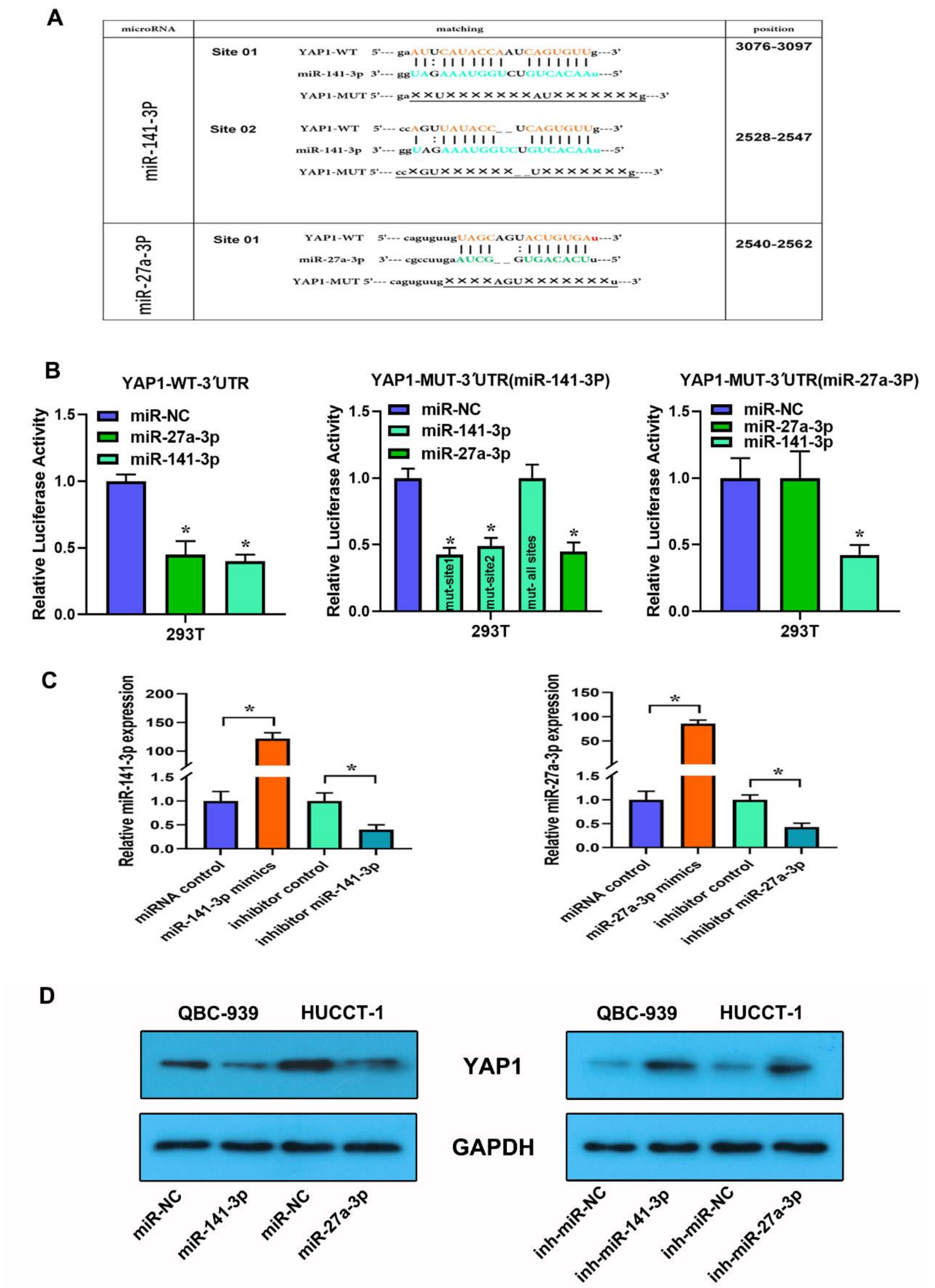

Supplementary Figure 3 miR-141-3p and miR-27a-3p can bind and regulate YAP1 expression. **(A)** The schematic of the binding sites about miR-141-3p and miR-27a-3p in the 3’ UTR of YAP1. **(B)** Luciferase activity in 293T cells co-transfected with miR-141-3p and miR-27a-3p and the corresponding luciferase reporters that were empty or contained YAP1 or a mutant transcript. The data are presented as the relative ratio of firefly luciferase activity to Renilla luciferase activity. Data are mean±SD. *P < 0.05. The experiment was repeated three times. **(C)** The expression levels of

miR-141-3p and miR-27a-3p after overexpression or inhibition were detected by RT-PCR in ICC cells. **(D)** Western blotting was performed to detect the effects on YAP1 protein levels after miR-141-3p or miR-27a-3p was overexpressed or inhibited in ICC cells.
