# Supplementary material for: Long Non-Coding RNA-PAICC Promotes the Tumorigenesis of Human Intrahepatic Cholangiocarcinoma by Increasing YAP1 Transcription
Source: Front Oncol. 2021 Jan 8;10:595533. doi: 10.3389/fonc.2020.595533 (PMC7856545; doi:10.3389/fonc.2020.595533)
Supplement: Supplementary file 5 [file Table_1.docx]

## Appendix Table S1. Sequence information used in this study

| **Sequence name** | **Sequence** |
| --- | --- |
| shRNA1-PAICC sense | 5′-GCAGTAGATAACCAACATT-3′ |
| shRNA2-PAICC sense | 5′-GCTGCAAACTGGCATGAAT-3′ |
| shRNA3-PAICC sense | 5′-CCTTGCTGAGCTTGCTTAT-3′ |
| shRNA-NC | 5′-TTCTCCGAACGTGTCACGT-3′ |
| LncRNA-PAICC Probe  For FISH | 5′-AATGTTGGTTATCTACTGC-3′(5'-Cy3) |
