# Supplementary material for: Long Non-Coding RNA-PAICC Promotes the Tumorigenesis of Human Intrahepatic Cholangiocarcinoma by Increasing YAP1 Transcription
Source: Front Oncol. 2021 Jan 8;10:595533. doi: 10.3389/fonc.2020.595533 (PMC7856545; doi:10.3389/fonc.2020.595533)
Supplement: Supplementary file 6 [file Table_2.docx]

## Appendix Table S2. Primers used for real-time PCR

| **Primer names** | **sequences** |
| --- | --- |
| LncRNA-PAICC forward | AGCCTTGTGCATTCCTGACA |
| LncRNA-PAICC reverse | CGCTACAGTCCCTGGTAAGC |
| CRYM-AS1 forward | GGCGGCGATGTATTGCTTTA |
| CRYM-AS1 reverse | CAGGCTGAGCAGCAAAATCG |
| RP11-57A19.2 forward | ATAAACTTGGGCCTGGGTGG |
| RP11-57A19.2 reverse | TTCTATCCGGCGAGCAGTTC |
| CTD-2132N18.2 forward | GGCGTCAAGGTGGAGTTAGA |
| CTD-2132N18.2 reverse | ATCCTCCTTTGCCATGCAGT |
| RP11-74O3.3 forward | CATCCGTGCACATGCATCAC |
| RP11-74O3.3 reverse | CTCATCCCCACTTGTGGCTC |
| YAP1 forward | TGCGTAGCCAGTTACCA |
| YAP1 reverse | GGTGCCACTGTTAAGGA |
| β-Actin forward | GGGAAATCGTGCGTGACATTAAG |
| β-Actin reverse | TGTGTTGGCGTACAGGTCTTTG |
| U6 forward | CTCGCTTCGGCAGCACA |
| U6 reverse | AACGCTTCACGAATTTGCGT |
| GAPDH forward | AGAAGGCTGGGGCTCATTTG |
| GAPDH reverse | AGGGGCCATCCACAGTCTTC |
| has-miR-141-3p-F  hsa-miR-141-3p-RT | CGCCTAACACTGTCTGGTAA  GTCGTATCCAGTGCAGGGTCCGAGGTATTCGCACTGGATACGACCCATCT |
| miR-27a-3p-F  hsa-miR-27a-3p-RT | GCGCTTCACAGTGGCTAAG  GTCGTATCCAGTGCAGGGTCCGAGGTATTCGCACTGGATACGACGCGGAA |
| has-miR-150-3p-F | CTGGTACAGGCCTGGG |
| Tniverse-R | ATATGTGCAGGGTCCGAGGT |
